# A midline Becker's nevus

**DOI:** 10.1016/j.jdcr.2025.05.037

**Published:** 2025-06-25

**Authors:** Connor A. Sheehan, Saira N. Agarwala, Sylvia Hsu

**Affiliations:** Department of Dermatology, Temple University Lewis Katz School of Medicine, Philadelphia, Pennsylvania

**Keywords:** Becker's nevus, Becker's nevus syndrome, confluent and reticulated papillomatosis of Gougerot and Carteaud, cutaneous hamartoma

## Introduction

Becker's nevus (BN) is a cutaneous hamartoma that presents as a unilateral, hyperpigmented patch, sometimes with associated hypertrichosis.^1^ The genetic etiology and pathogenesis of BN are not clear; it is currently considered a sporadic condition.^2^ Histopathology of these lesions shows acanthosis, basilar hyperpigmentation, and occasionally, hyperplasia of the underlying smooth muscle.^3^ There is no established treatment.^1^ Clinical appearance, morphology, and distribution often raise suspicion for BN, yet there have been an increasing number of variants and associations published in recent years. A recent article delineated the distribution of BNs as sparing the midline chest.^4^ In this report, we present a case of BN that mimicked confluent and reticulated papillomatosis (CARP) of Gougerot and Carteaud because of its midline chest distribution.

## Case report

**Fig 1:**
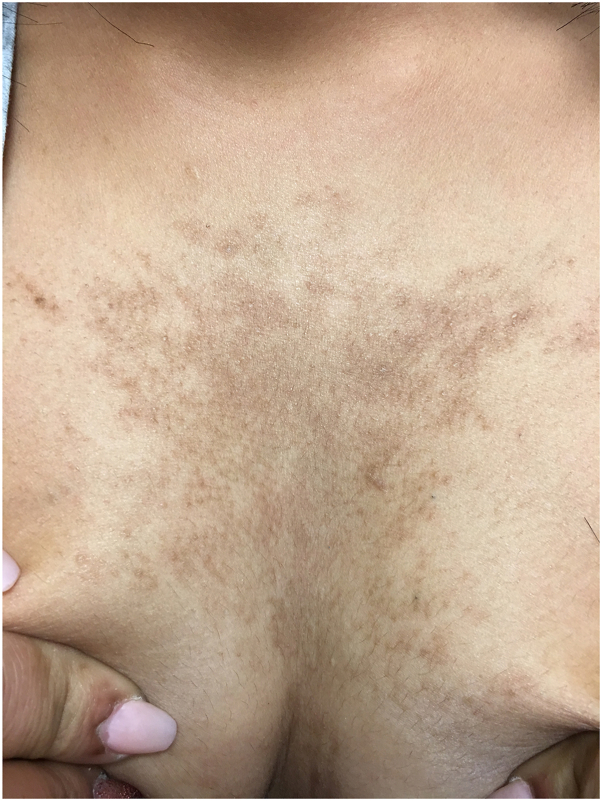
Clinical image of the midline patch at initial presentation.

**Fig 2:**
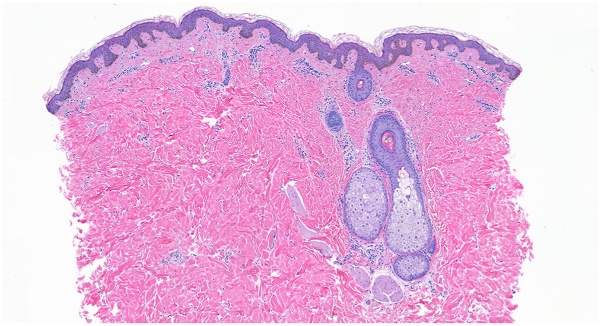
Histopathology showed accentuation of pigment at the tips of rete ridges and a subtle increase in smooth muscle, consistent with Becker's nevus (H&E 100×).

A 21-year-old woman with a past medical history of diabetes mellitus presented to our dermatology clinic with a reticulated hyperpigmented patch on the midline chest (Fig 1). Her neck, back, and axillae were unaffected. At the time, the patient was given a diagnosis of CARP of Gougerot and Carteaud and was started on 100 mg of minocycline twice daily for 12 weeks. The patient returned to our clinic over 7 years later, at which point the midline patch was identical to her initial presentation. The patient revealed that the area of hyperpigmentation had been present and stable since 13 years of age. After further examination, a diagnosis of BN was made clinically. To confirm our clinical diagnosis, a 4-mm punch biopsy was performed. Histopathology showed no evidence of CARP, but rather it displayed accentuation of pigment at the tips of elongated and clubbed rete ridges and smooth muscle bundles in the dermis, consistent with BN (Fig 2).

## Discussion

We present a unique case of a BN presenting in the midline chest, representing an update to the existing literature regarding the distribution of these lesions. It is common to encounter cases of BNs involving portions of the intermammary region; however, most reported anterior chest lesions involve the shoulder and/or are associated with underlying breast hypoplasia, and none are perfectly midline.^5^, ^6^, ^7^ Our patient's lesion was initially confused clinically for CARPs because of the midline location and the reticulated pattern at the edge of the lesion. However, the reticulated pattern classically seen in CARP has also been described in BNs by Criscione and Telang^8^ and represents an important clinical pearl for a diagnosis of BN.

**Fig 3:**
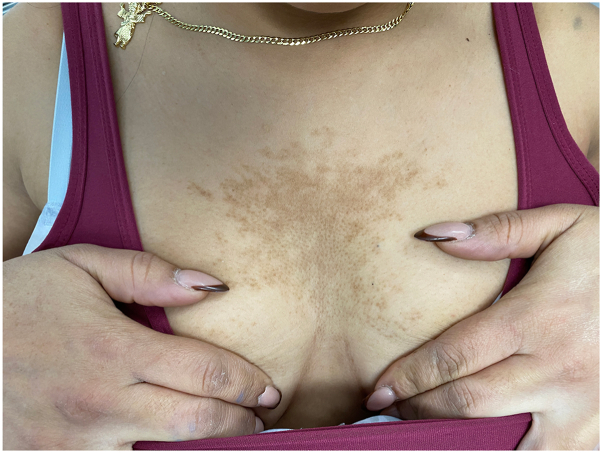
Clinical image of the midline patch 7 years later.

Pathology of the lesion did not show CARP, nor did it behave clinically like CARP. The lesion showed no change in response to oral minocycline and remained perfectly stable over the course of 7 years (Figs 1 and 3). Biopsy can distinguish BN from other dermatologic conditions should the clinical picture generate a differential diagnosis, although the reliability of obtaining a section that displays the smooth muscle hamartoma is difficult in clinical practice. The patient's lesion dates back to her early teens, which is consistent with the proposed sporadic postzygotic mutation mechanism of solitary BN formation due to lethal mutations in the *ACTB* gene encoding beta-actin within pilar muscle cells.^1^^,^^9^ It is hypothesized that the appearance of these lesions occurs near puberty due to increased androgen receptor expression within BN and thus phenotypic change.^1^ It is clinically relevant to distinguish CARP from BN, as their natural history, associated findings, and treatment are entirely divergent. BN is not a concerning finding; however, it may be associated with other cutaneous and extracutaneous anomalies, including underlying hypoplasia.^2^^,^^10^ Treatment of BN is indicated for cosmetic purposes and is typically targeted at reducing hyperpigmentation via topical flutamide, topical glycolic acid, dermabrasion, or laser therapy, despite mixed results.^1^ Our case illustrates the importance of knowing that BN can present as a midline patch on the chest. Initially, we had misdiagnosed the patient as having CARP, since the existing literature states that midline BN does not occur.

## Conflicts of interest

None disclosed.
